# Integrating technology into a successful apomorphine delivery program in Thailand: a 10-year journey of achievements with a five-motto concept

**DOI:** 10.3389/fneur.2024.1379459

**Published:** 2024-04-05

**Authors:** Onanong Phokaewvarangkul, Nithinan Kantachadvanich, Vijittra Buranasrikul, Kanyawat Sanyawut, Saisamorn Phumphid, Chanawat Anan, Roongroj Bhidayasiri

**Affiliations:** ^1^Chulalongkorn Centre of Excellence for Parkinson’s Disease & Related Disorders, Chulalongkorn University and King Chulalongkorn Memorial Hospital, Bangkok, Thailand; ^2^The Academy of Science, The Royal Science of Thailand, Bangkok, Thailand

**Keywords:** Parkinson’s disease, apomorphine, infusion therapy, daycare service, technology

## Abstract

**Introduction:**

Apomorphine, a potent dopamine agonist, is a therapeutic option for patients with Parkinson’s disease and motor fluctuations. However, the adoption of and adherence to this therapy have been limited by the need for complex delivery devices and specialized care as well as resource consumption, posing challenges for new physicians. Thailand is a unique example of a developing nation that has successfully implemented and continued the use of this therapy by employing cooperative technology that has dramatically enhanced apomorphine delivery services.

**Methods:**

Establishing apomorphine delivery services requires significant resources and step-by-step solutions. We began our services by implementing various strategies in three chronological stages: the initial stage (2013–2015), intermediate stage (2016–2019), and current stage (2020–present), each presenting unique challenges. Together, we also implemented a proposed set of five mottos to strengthen our apomorphine delivery service. Using additive technology, we developed a patient registry platform that combined electronic data acquisition, video and remote monitoring using wearable sensors, and in-house mobile applications to support our service.

**Results:**

At the initial stage, we assembled a team to enhance the efficacy and confirm the safety of apomorphine treatment in our hospital. At the intermediate stage, we expanded our apomorphine delivery services beyond just the patients at our hospital. We supported other hospitals in Thailand in setting up their own apomorphine services by educating both physicians and nurses regarding apomorphine therapy. With this educational undertaking, increased apomorphine-related knowledge among medical professionals, and a greater number of hospitals providing apomorphine services, an increasing number of patients were administered apomorphine in subsequent years. Currently, we are providing effective apomorphine delivery to improve patient outcomes and are seamlessly integrating technology into clinical practice. Incorporating integrative technologies in our apomorphine delivery program yielded positive results in data collection and support throughout patient care, in tracking patients’ statuses, in the long-term use of this treatment, and in increasing medication adherence rates.

**Conclusion:**

This perspective paper describes how technology can help provide supportive healthcare services in resource-constrained environments, such as in Thailand, offering a step-by-step approach to overcoming several limitations. The valuable insights from our 10-year journey in successfully integrating technology into apomorphine delivery services can benefit new physicians seeking to replicate our success.

## 1 Introduction

The use of apomorphine, a non-ergoline dopamine agonist, has emerged as an effective option for the management of severe motor fluctuations in patients with Parkinson’s disease (PD) (1–3). Apomorphine therapy offers rapid and reliable relief to patients with PD-related motor complications. Its effectiveness is similar to that of other device-aided therapies (DATs), including deep brain stimulation (DBS) and levodopa-carbidopa intestinal gel infusion (LCIG) (4). Compared to other DAT modalities, continuous subcutaneous apomorphine infusion (CSAI) is more advantageous for treatment initiation owing to its lower invasiveness and reduced device complexity (5, 6). Moreover, according to the United Kingdom National Institute for Health and Care Excellence guidelines, apomorphine should be offered before other DAT options that involve surgical intervention (7).

However, establishing apomorphine delivery services requires considerable resource allocation owing to its intricate and time-consuming processes, including the need for an expert multidisciplinary team to manage the device, provision of individualized patient care, and the requirement of a specialized center for apomorphine titration initiation (8). Moreover, PD nurse specialists (PDNSs) play a pivotal role in supporting patients and ensuring the success of such services by overseeing apomorphine use, handling needles and devices, and managing medication adjustments on behalf of neurologists (8). The setting for treatment initiation is also important, with most patients requiring hospital-initiated apomorphine therapy to ensure precise drug delivery (9, 10). Overcrowded hospitals remain a challenge, delaying the commencement of such therapies in new patients (11); hence, home-initiated apomorphine therapy has emerged as a solution in this context. Nevertheless, the increasing demand for PDNSs poses a new issue for introducing this treatment in clinic or at home (10, 11). Therefore, it is necessary to establish specialized centers with proper equipment and trained interdisciplinary staff (9, 12). As a consequence, newly trained physicians aspiring to adopt this treatment regimen may encounter difficulties due to its complexity and staff demands. Therefore, apomorphine is underutilized in developing countries (9). Thailand is one of the few developing countries that has initiated and maintained a specialized apomorphine delivery service from 2013 until now.

Establishing an apomorphine delivery service involves several challenges, including managing treatment methods and associated side effects, addressing patient concerns, and resolving payment-related issues (12). This perspective paper offers insights from our real-life experiences regarding the implementation of apomorphine treatment in a challenging environment. Although we acknowledge that no single treatment is perfect, our aim was to achieve continuous daily improvement.

## 2 Methods and results

**Figure 1:**
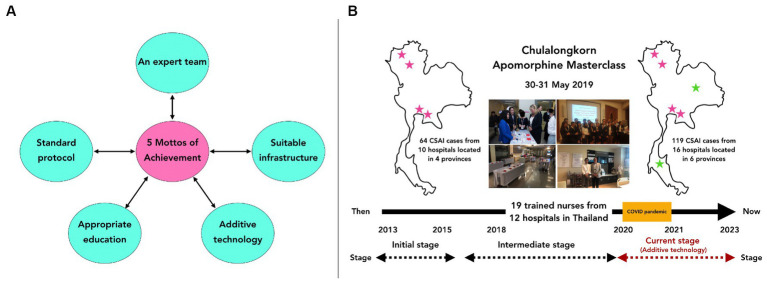
**(A)** The five mottos of achievement of our apomorphine service, which is composed of 5 components including; (1) an apomorphine expert team, (2) standard protocols for proper patient selection, (3) suitable infrastructure and facilities, (4) appropriate education and training, and (5) additive technologies to enhance patient outcomes. **(B)** Between 2013 and 2023, a total of 119 patients in Thailand received CSAI. Before 2019, 64 patients underwent CSAI in 10 hospitals located in 4 provinces. However, after the development of training courses for physicians and nurses since 2019, including the ‘Apomorphine Masterclass’ and the ‘Experienced Nurse Training for Apomorphine Therapy,’ we observed an increasing number of hospitals in various provinces throughout Thailand beginning to offer apomorphine services. This led to a rise in the number of patients receiving CSAI each year, despite the challenges posed by the coronavirus-2019 pandemic from 2019 to 2021, which restricted patient access to apomorphine services due to lockdown situations in Thailand.

The Chulalongkorn Center of Excellence for Parkinson’s Disease and Related Disorders (ChulaPD; www.chulapd.org) is one of the largest referral centers in Thailand for treating patients with PD (12). We initially introduced apomorphine use as one of the three treatment modalities for PD in our DAT clinic, alongside DBS and LCIG use. At the beginning of the apomorphine delivery service setup, several problems with lacking manpower and dealing with device difficulty were identified, requiring step-by-step solutions. Our apomorphine delivery service has evolved over the last 10 years. We proposed five components in mottos as necessary for establishing a successful apomorphine delivery service using a patient-centric strategy (Figure 1A). These components are: (1) an apomorphine expert team, (2) standard protocols for proper patient selection, (3) suitable infrastructure and facilities, (4) appropriate education and training, and (5) additive technologies to enhance patient outcomes. However, completing all these steps may not be easy, and our incremental approach highlights the importance of time and a stepwise approach to achieving success.

### 2.1 Stages for developing our apomorphine delivery services: initial, intermediate, and current stages

We developed our apomorphine delivery services in three stages: the initial, intermediate, and current stages. We utilized CSAI, as this was the sole modality for apomorphine treatment available in Thailand in 2023. By employing additive technology, we maintained the standard of apomorphine treatment while reducing manpower consumption and enhancing the ability to record data for further studies.

#### 2.1.1 Initial stage of our apomorphine service (2013–2015)

In this stage, we implemented the first component––an apomorphine expert team, and focused primarily on assembling a team to enhance the efficacy and safety of apomorphine treatment. Unfortunately, only a small percentage of centers have multidisciplinary teams, and a considerably small number of centers have PDNSs (13). Running an apomorphine delivery service may not require a large team; however, the team should be efficient. Hence, we piloted a program with a small group of physicians and nurses with clinical expertise, as there were no PDNSs. Subsequently, general nurses were trained to become experienced nurses to support the team by administering CSAI, closely monitoring patient responses, and providing educational interventions to patients and caregivers (8, 14). At this stage, we began administration of in-hospital apomorphine therapy to ensure precise drug delivery and recruit enough patients with PD. The benefits and efficacy of apomorphine administration were pronounced, giving our team members confidence to practice their skills for this drug treatment.

#### 2.1.2 Intermediate stage of our apomorphine service (2016–2019)

In this stage, we expanded our apomorphine delivery services beyond patients at our hospital. We began to function as a referral center. Moreover, we supported other hospitals in Thailand in setting up their own apomorphine services by educating both physicians and nurses to understand and manage apomorphine treatment. To expand the apomorphine service at our hospital and other hospitals, we implemented three additional components. First, we developed standard protocols for proper patient selection by adapting our service to follow the established protocol for patient selection and treatment (5, 15). Suitable candidate selection ensured enhanced patient adherence and treatment safety (16).

Second, we developed suitable infrastructure and facilities, believing that a suitable healthcare environment would promote positive outcomes and help to maintain long-term treatment. However, as King Chulalongkorn Memorial Hospital is the leading tertiary referral hospital in Thailand with a capacity of 1,500 beds, serving over 1.5 million outpatients per year (17), hospital overcrowding remained our biggest challenge, delaying the commencement of treatment for new patients. To overcome this problem, our apomorphine delivery service was initiated in an outpatient setting with daycare services, providing short-term opportunities to increase patient admissions. The outpatient clinic with daycare service was well organized and equipped to support efficient treatment administration even during emergencies (18). Moreover, creating a functional, patient-friendly clinic was crucial, and our floor plan for the daycare room was developed to ensure functionality.

Third, we implemented appropriate education and training. Supporting education for patients and caregivers is vital for the long-term continuation of apomorphine treatment. In addition to helping new patients understand and accept the invasive treatment, we supported proper handling of device complexities. This contributed to realistic expectations and a more positive patient experience with the advanced therapy (19). Appropriate education and training are cornerstone therapeutic advantages, and building a multidisciplinary care team for apomorphine treatment is essential. Consequently, we conducted comprehensive apomorphine-related training sessions tailored to medical professionals, including physicians, nurses, and other allied health workers. These personnel came from various hospitals to gain experience regarding apomorphine and gain support in establishing apomorphine services at their local hospitals and recruiting more patients. These sessions have been held through in-person and virtual meetings since 2019, including the **“Apomorphine Masterclass”** and the **“Experienced Nurse Training for Apomorphine Therapy”** (Figure 1B). Through these educational programs, we actively maintain a collaborative network with all medical professionals who participated in these meetings to ensure their proficiency and comprehension of apomorphine-based PD treatment in real clinical practice. Moreover, since our educational program was implemented, an increasing number of hospitals in various provinces throughout Thailand have begun to offer apomorphine services, with a growing number of patients receiving apomorphine each year, even from 2019 to 2021, when the coronavirus-2019 pandemic restricted the number of patients able to access of apomorphine service due to the lockdown situation in Thailand (Figure 1B). This information confirms the value of our attempt to support and enhance the apomorphine service in Thailand.

#### 2.1.3 Current stage of our apomorphine service (2020–present)

**Figure 2:**
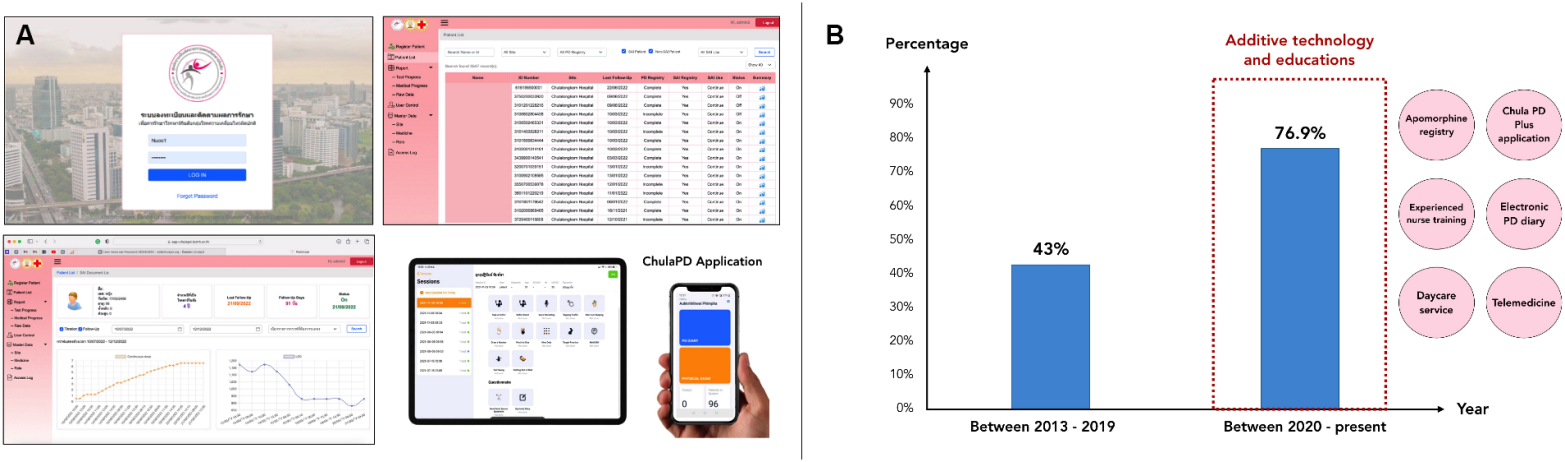
**(A)** Depicts a series of figures illustrating both the apomorphine registry platform on the website and the in-house ChulaPD Plus application available on mobile phones and tablets. This platform serves the purpose of recording data from patients undergoing apomorphine treatment in hourly increments of dosage. Additionally, it can represent outcomes or improvements in a graph for easy follow-up or understanding. **(B)** Among patients initiated on CSAI at our center between 2013 and 2019, 42% of those who started and continued treatment for over 12 months were found to have done so. Interestingly, following the implementation of educational programs and technology integration for patient progress tracking, a notable increase in the proportion of patients was observed, with 76.9% of patients who started the CSAI treatment since 2020 continuing treatment over 12 months. Additionally, additive technology and educations have been conducted mainly since 2020, including web-based apomorphine registry, ChulaPD Plus application, electronic PD diary, daycare service, telemedicine, and experienced nurse training.

In the current stage, we implemented the final component, which was additive technologies to enhance patient outcomes. These technologies enhanced apomorphine delivery and improved patient outcomes. During this stage, we not only achieved effective apomorphine delivery but also seamlessly integrated technology into clinical practice (12). We developed a precise delivery method to ensure effective and safe apomorphine administration to patients with PD, and technology was used to enhance our program. A game-based mobile application, ChulaPD Plus®, was developed in-house to objectively assess patient performance (Figure 2A). An electronic health record systems also facilitated data tracking and patient management (20). An In-house, web-based apomorphine registry was developed to track patient progress during the titration and follow-up periods (Figure 2A). In addition, we also developed an electronic PD diary to replace a traditional paper-based diary, which is more accessible and has better readability for patients. This electronic PD diary is also helpful for patient to communicate better with medical professionals (Supplementary Figures S1A,B). Moreover, the daily additional use of wearable sensors for objective detecting may be employed for addressing particular patient concerns, such as nocturnal akinesia, referred to as ‘nocturnal device’ (Supplementary Figure S1C).

Furthermore, the application of machine learning in technology-based monitoring triggers the identification of suitable candidates, optimal dose determination, and potential adverse event detection.

Maintaining patient connections and conversations ensures treatment understanding, and the team supports patients if any problems or concerns arise. These elements are vital for supporting long-term usage of this treatment. For emergency cases, we offer a call-in LINE® program or telemedicine option for urgent consultation directly with our experienced nurse. The group chat in Line® program, also provide additional educational tools related to apomorphine, massage and needle handling techniques, and upcoming patient support group meetings. We have applied these additive technologies to the care of patients at our center receiving apomorphine since 2020. The differences between traditional apomorphine delivery service initiation and the use of additive technology in apomorphine delivery services are shown in Supplementary Table S1.

### 2.2 Technology-based healthcare services for apomorphine-treated patients with PD

With additive technology, standard apomorphine therapy can be continued while reducing manpower consumption and enhancing the ability to record data for further studies. Technology aids in the selection of suitable candidates and supports teams during apomorphine titration, treatment maintenance, and remote monitoring of patients. Additionally, the retrieved information from technology-based tracking supports further analysis, as seen in our previous publications (21, 22). Furthermore, it provides support in many aspects of apomorphine management, both in the short-term and the long-term, as outlined below.

#### 2.2.1 Technology helps select suitable candidates

Specific eligibility criteria for apomorphine treatment vary according to regional guidelines, but the final patient selection also depends on the physician’s judgment and patients’ circumstances (10). Although these criteria are employed in routine patient selection in most specialized centers, this approach does not guarantee long-term apomorphine use; hence, the criteria are decreasingly used (22, 23). In a recent publication based on our apomorphine registry, we identified three major predictors for apomorphine discontinuation: (1) absence of full-time caregivers, (2) patients who achieved a daily off-hour reduction of <3.5 h after apomorphine titration, and (3) patients with high non-motor symptoms questionnaire scores (i.e., ≥9.5 points) at the time of CSAI titration (21). Therefore, we suggest considering these factors as red flags when evaluating treatment responses to CSAI and making decision about the long-term use of this treatment or closely monitoring these patients during follow-up. Although some patients may show positive benefits from CSAI, those patients who exhibit these factors are more likely to discontinue CSAI therapy in the long-term. Additionally, to accurately evaluate off-hour or dyskinesia hours, an electronic PD diary may prove beneficial, providing better readability for interpreting patients’ symptoms by medical professionals (24).

#### 2.2.2 Technology helps support and track patient data during apomorphine titration

Most patients who underwent apomorphine treatment in our center received 10–12 days of titration doses in an outpatient clinic with daycare service, and experienced nurses were instrumental in educating and supporting patients and caregivers in administration techniques, pump handling, side effects, and strategies to effectively manage daily activities (8). During titration, experienced nurses educated the patients, assessed their understanding of the treatment method, and provided step-by-step hands-on device control advice (8). The patients’ video will be recorded in every time-point of apomorphine increment to ensure patient’s mobility and improvement. Experienced nurses also tracked patient improvement using an in-house, web-based apomorphine registry, a game-based mobile application, an electronic PD diary, which are less time consuming and require no significant manpower. Data collection was longitudinally completed using these technology-based electronic patient records and electronic rating scales, which are part of our apomorphine registry. Furthermore, technology was utilized to facilitate nighttime patient monitoring; for example, ‘nocturnal devices’ which are wearable sensors, were employed to capture the improvement of nocturnal akinesia after nighttime apomorphine infusion (25). These methods are beneficial for long-term patient follow-up and support future studies.

#### 2.2.3 Technology helps maintain patient treatment and remote monitoring

Follow-up and long-term monitoring of the adverse events of apomorphine infusion in patients with PD are crucial to ensuring the safety and efficacy of treatment over time. Regular assessments, communication with healthcare professionals, and adjustments to the treatment plan help manage potential side effects and maintain optimal outcomes (16). Owing to device complexities and possible side effects, patients may have doubts, issues, and misunderstandings regarding treatment. Common adverse events associated with apomorphine infusion include nausea and vomiting, orthostatic hypotension, subcutaneous nodules or skin reactions, dyskinesia, and neuropsychiatric symptoms (26). Maintaining patient connection and conversation ensures treatment understanding, and the team supports patients if any problems or concerns arise. Moreover, remote monitoring is helpful to identify adverse events that can be observed visually, such as subcutaneous nodules or skin reactions at the infusion site that may lead to apomorphine discontinuation. Experienced nurses are able to teach patients online to maintain good hygiene, correctly apply needle injection and massage methods, and change the infusion site daily to prevent adverse events. Moreover, patients are encouraged to consult a physician in the case of severe skin conditions or infections (8). Most patients took photos of their abdomen to send to the team, ensured a good angle of needle injection, examined themselves for severe nodules, and received encouragement from the team, which enhanced their long-term apomorphine use.

The benefits of a supportive educational program and integrating technology using these platforms to track patients’ information and progression not only support data collection and future studies but also support patients in long-term treatment continuation by tracking any concerns or side effects of treatment and resolving those problems. The impact of our work has been found by examining the percentage of patients who remained on CSAI therapy for more than 12 months, a period during which most patients would discontinued treatment if they encountered related problems (21, 27). Since 2013, a total of 119 patients in Thailand were treated with apomorphine infusion, of whom 80 (67.2%) were patients at King Chulalongkorn Memorial Hospital. Among patients initiated on CSAI at our center between 2013 and 2019, 42% of patients who started and continued it for over 12 months were found. Interestingly, following the implementation of educational programs and technology integration for CSAI patients, a notable increase in the proportion of patients was observed, with 76.9% of patients who started CSAI treatment since 2020 continuing treatment for over 12 months (Figure 2B).

## 3 Discussion

A decade ago, we established an apomorphine delivery service for Thai patients with PD. We started with a small but efficient team, and we have achieved good efficacy through longitudinal follow-up. The first patient to undergo CSAI in Thailand was treated at our center in 2013; thereafter, outpatient apomorphine delivery and experienced nurse training programs were initiated in 2015 (8). Currently, our apomorphine delivery service serves the highest number of CSAI patients in Thailand (12). To address issues of treatment availability and reimbursement, establishing an apomorphine therapy collaboration network helped create a practical referral system to assist with patient needs, provide crucial therapy-related information, and open a line of communication between medical specialists in our centers and those in nearby hospitals. This collaborative network also helped improve the understanding of physicians and nurses regarding long-term DAT for PD (12). These examples illustrate the establishment of a successful outpatient apomorphine delivery system in a clinical setting. Our 10-year experience enables us to highlight challenges and successes in PD management.

We demonstrated that technology can be adopted in different stages of a patient’s journey with apomorphine treatment, and the benefits include patient selection, monitoring responses to treatment, detection of adverse events, and prediction of unfavorable outcomes. Acknowledging the ongoing global changes in technology and digital biomarkers, our apomorphine registry platform includes game-based tests within a mobile application to objectively assess patient performance. We are currently conducting a pilot study using machine learning to identify the predictive factors for improved outcomes in apomorphine-treated patients. In a changing world, changes in apomorphine delivery methods to alleviate issues related to needle phobia and pump complexity are necessary and may be resolved with new technologies, such as transdermal patches, nanoneedles, and non-injection administration methods. Moreover, artificial intelligence may be employed to identify subcutaneous nodules in images. Previous studies have shown that artificial intelligence, which can help clinicians efficiently tailor treatment, has potential benefits for wound assessment (28, 29). In the future, patients’ information may be used with integrative technologies such as artificial intelligence for early identification and treatment of apomorphine-related side effects. This technology would help reduce manpower, enhance objective data recording, and simultaneously improve patient care.

There are several limitations to consider in our perspective paper. Firstly, it’s important to note that most of the data we collect are from our center, so they may not fully represent the demographic characteristics of all apomorphine patients in our country, which could affect the diversity of discontinuation of treatment. Secondly, many patients who discontinue CSAI were initiated on the treatment in the past, particularly during the period from 2013 to 2019. Given the length of time since initiation, it’s challenging to solely attribute the recent benefits of additive technology to support the use of apomorphine. However, providing data on the higher percentage of patients who remained on CSAI therapy for more than 12 months after receiving supportive educational programs and integrating technology since 2020 would offer a more unbiased and objective assessment of the true impact of our proposed interventions. Moreover, our outpatient service yielded positive results that strongly support the continuation of this treatment in the long-term, including the identification of suitable candidates, tracking possible side effects, recording longitudinal data, and promoting patient well-being and education. Hence, our findings will enhance long-term patient management and support future studies. In summary, our strategy offers valuable insights into overcoming obstacles associated with the initiation of apomorphine therapy in outpatient clinic using daycare services. Collaboration and networking with other medical institutions can foster robust support systems, thereby facilitating the expansion and success of apomorphine delivery clinics.

In the future, apomorphine treatment can be improved to enhance the quality of life of patients with PD. One key direction is the use of home-based treatment with integrated technology, allowing for real-life assessments and reducing the need for frequent hospital visits, especially during severe OFF stages. Various transfusion methods, such as microneedles and transdermal patches, show promise as drug delivery options. Utilizing technology for remote monitoring and telemedicine can provide more personalized and convenient care. To achieve this vision, continued research, development, and collaboration among healthcare providers, pharmaceutical companies, and technology experts are essential.

## Data availability statement

The original contributions presented in the study are included in the article/Supplementary material, further inquiries can be directed to the corresponding author.

## Ethics statement

The studies involving humans were approved by Chulalongkorn University Institutional review board. The studies were conducted in accordance with the local legislation and institutional requirements. The participants provided their written informed consent to participate in this study.

## Author contributions

OP: Conceptualization, Methodology, Supervision, Writing – original draft, Writing – review & editing. NK: Methodology, Writing – review & editing. VB: Methodology, Writing – review & editing. KS: Methodology, Writing – review & editing. SP: Methodology, Writing – review & editing. CA: Methodology, Writing – review & editing. RB: Conceptualization, Funding acquisition, Methodology, Resources, Supervision, Writing – review & editing.
