# Supplementary figures and images for: Integrating technology into a successful apomorphine delivery program in Thailand: a 10-year journey of achievements with a five-motto concept
Source: Front Neurol. 2024 Apr 5;15:1379459. doi: 10.3389/fneur.2024.1379459 (PMC11026563; doi:10.3389/fneur.2024.1379459)

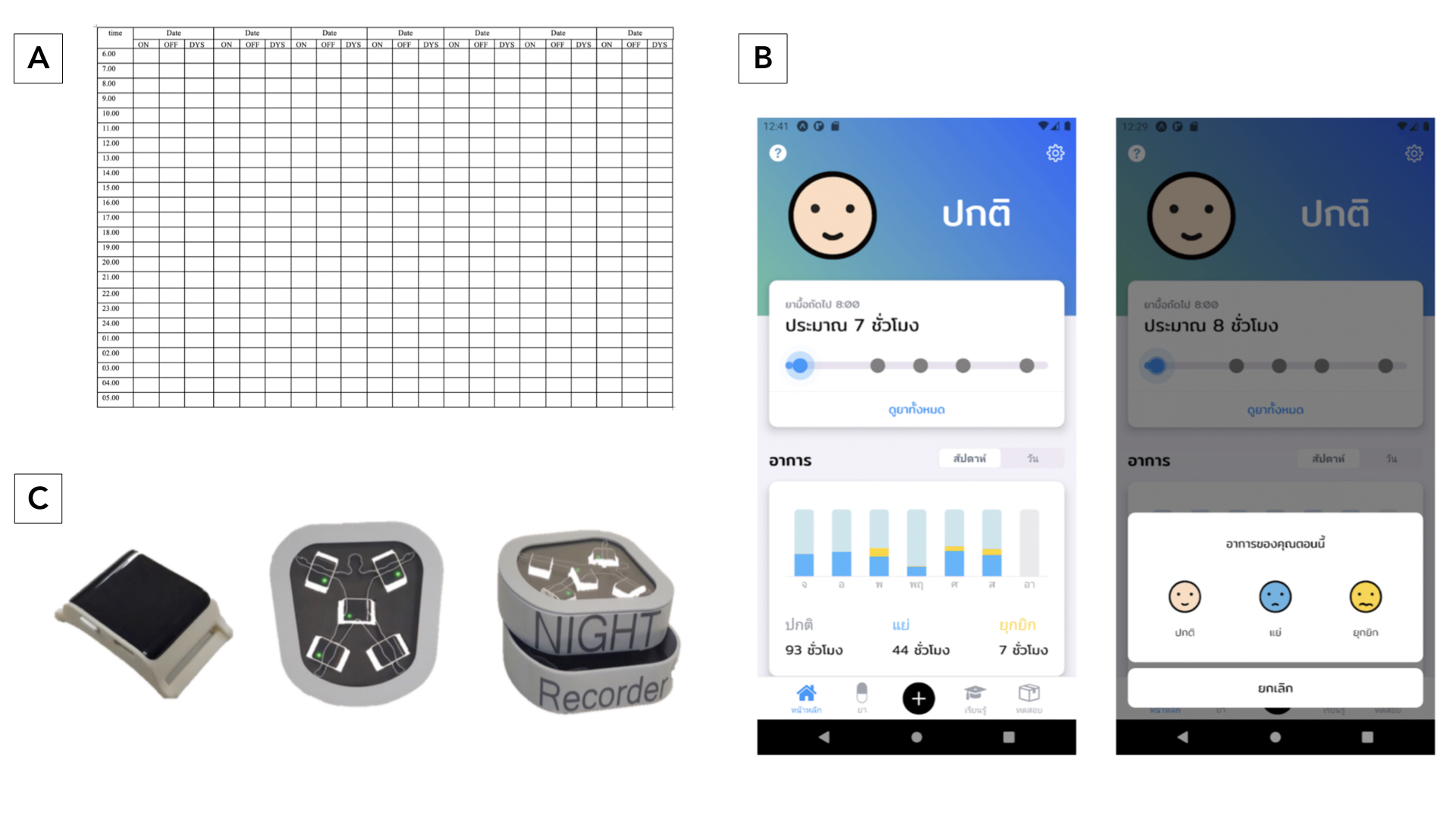

Supplement: Supplementary file 1 [file Image_1.JPEG]
