# Supplementary material for: Integrating technology into a successful apomorphine delivery program in Thailand: a 10-year journey of achievements with a five-motto concept
Source: Front Neurol. 2024 Apr 5;15:1379459. doi: 10.3389/fneur.2024.1379459 (PMC11026563; doi:10.3389/fneur.2024.1379459)
Supplement: Supplementary file 2 [file Table_1.DOCX]

Supplementary Table 1 shows the difference between the traditional way of starting an apomorphine service and additive technology in apomorphine services.

| **Traditional ways** | **Additive technology ways** |
| --- | --- |
| High consume manpower | Less consume manpower |
| Mainly subjective evaluation | Both subjective and objective evaluations |
| Basic rating scale | Comprehensive rating scale |
| Paper-based documents | Paper-less documents |
| Paper-based Parkinson’s disease diary | Mobile application for Parkinson’s disease diary |
| Paper note for patient’s symptoms changes | In-house web-based Apomorphine registry platform for recording patients’ symptoms changes |
| Occasional video recording | Protocols of video monitoring for each increment of apomorphine dosage |
| Basic mobility tests such as finger tapping and walking tests | In-house ChulaPD application for objective evaluation of dexterity and mobility tests |
| No wearable sensors | Additional use of wearable sensors for objective detecting particular patient’s concerns such as nocturnal akinesia, tremor, etc. |
